# More than 30 Years of POSSUM: Are Scoring Systems Still Relevant Today for Colorectal Surgery?

**DOI:** 10.3390/jcm13010173

**Published:** 2023-12-28

**Authors:** Florian Bürtin, Tobias Ludwig, Matthias Leuchter, Alexander Hendricks, Clemens Schafmayer, Mark Philipp

**Affiliations:** 1 Department of General, Visceral, Thoracic, Vascular and Transplantation Surgery, University Medical Center Rostock, 18057 Rostock, Germanyclemens.schafmayer@med.uni-rostock.de (C.S.); mark.philipp@med.uni-rostock.de (M.P.); 2 Institute of Implant Technology and Biomaterials e.V., 18119 Rostock, Germany; matthias.leuchter@uni-rostock.de

**Keywords:** surgical audit, colorectal, cancer, POSSUM, P-POSSUM, CR-POSSUM

## Abstract

Background: The Physiological and Operative Severity Score for the enUmeration of Mortality and Morbidity (POSSUM) weights the patient’s individual health status and the extent of the surgical procedure to estimate the probability of postoperative complications and death of general surgery patients. The variations Portsmouth-POSSUM (P-POSSUM) and colorectal POSSUM (CR-POSSUM) were developed for estimating mortality in patients with low perioperative risk and for patients with colorectal carcinoma, respectively. The aim of the present study was to evaluate the significance of POSSUM, P-POSSUM, and CR-POSSUM in two independent colorectal cancer cohorts undergoing surgery, with an emphasis on laparoscopic procedures. Methods: For each patient, an individual physiological score (PS) and operative severity score (OS) was attributed to calculate the predicted morbidity and mortality, respectively. Logistic regression analysis was used to evaluate the possible correlation between the subscores and the probability of postoperative complications and mortality. Results: The POSSUM equation significantly overpredicted postoperative morbidity, and all three scoring systems considerably overpredicted in-hospital mortality. However, the POSSUM score identified patients at risk of anastomotic leakage, sepsis, and the need for reoperation. Logistic regression analysis demonstrated a strong correlation between the subscores and the probability of postoperative complications and mortality, respectively. Conclusion: Our results suggest that the three scoring systems are too imprecise for the estimation of perioperative complications and mortality of patients undergoing colorectal surgery in the present day. Since the subscores proved valid, a revision of the scoring systems could increase their reliability in the clinical setting.

## 1. Introduction

In recent decades, the self-conception of medicine has changed from a traditional, experienced-based tenet to an evidence-based science whose methods and results are objectifiable and reproducible. Since then, the medical community has witnessed the emergence of a plethora of scoring systems for the classification of the interindividual variability of health and disease, the evaluation of treatment efficiency, as well as for risk stratification of morbidity and mortality. The objective risk assessment for surgical procedures remains challenging since not only the patient’s individual health status but also the complexity of the procedure must be considered. However, an adequate evaluation of the perioperative risk is imperative to enable informed decision-making by the patient and effective resource allocation by the healthcare provider [1]. In 1991, Copeland et al. introduced the Physiological and Operative Severity Score for the enUmeration of Mortality and morbidity (POSSUM) for a surgical audit, weighting a physiological score, representing the patient´s overall health status, and a concomitant surgical severity score [2]. Whiteley et al. noted that the application of the original POSSUM equation on the patient population of two general surgery departments in Portsmouth, UK, overestimated mortality by a factor of two overall, and more specifically by a factor of six for patients with a low risk profile. As a result, the authors proposed a modified equation, called Portsmouth-POSSUM (P-POSSUM), with adjusted regression coefficients, leading to a more accurate estimate of perioperative mortality, especially in patients with a low risk profile [3]. Subsequently, the POSSUM methodology was adapted to the specific surgical specialties. Prytherch et al. modified the P-POSSUM equation to predict the mortality of the general vascular surgery population (V-POSSUM,) as well as the mortality of certain vascular conditions, like ruptured abdominal aortic aneurysm (RAAA-POSSUM) [4,5]. Additional modifications were established for orthopedic and esophagogastric surgery [6,7]. Since the POSSUM and P-POSSUM scoring systems underestimated the mortality of colorectal procedures regarding emergency surgeries and patients older than 80 years, Tekkis et al. proposed a simplified scoring system with a modified equation called the colorectal POSSUM (CR-POSSUM) [8].

The aim of this present study was to evaluate today’s significance of POSSUM, P-POSSUM, and CR-POSSUM in two independent colorectal cancer populations undergoing surgery, with an emphasis on laparoscopic procedures.

## 2. Materials and Methods

This study has been approved by the institutional review board of the University Medical Center Rostock (A2020–0193). The retrospective analysis included 485 patients undergoing surgery for colorectal cancer from two German tertiary centers covering the periods from 2002 to 2009 and from 2012 to 2019 (Figure S1). For each patient, an individual physiological score (PS) and operative severity score (OS) was attributed according to the scoring systems POSSUM [2], P-POSSUM [3] (Table 1), and CR-POSSUM [8] (Table 2).

Mortality for POSSUM, P-POSSUM, and CR-POSSUM, as well as morbidity for POSSUM, was calculated according to the original publications [2,3,6] (Table 3).

Contrary to the original publications, exact data for in-hospital mortality and morbidity was implied in place of the 30-day mortality and morbidity. Statistical analysis was performed with RStudio 1.4.1717-3 (RStudio, Inc., Boston, MA, USA) and GraphPad PRISM 9.0.0 (GraphPad Software, Inc., San Diego, CA, USA). For the comparison of dichotomous variables with contingency tables, the Fisher Exact and Chi2-Test were used, with *p* < 0.05 considered statistically significant. The comparison of the different POSSUM prognosis models was performed with the Kruskal–Wallis and Mann–Whitney-U Tests, with Dunn correction and *p* < 0.05 being statistically significant. The influence of the different variables on the postoperative outcome was determined by logistical regression. The predictive accuracy of POSSUM, P-POSSUM, and CR-POSSUM was tested by linear analysis for P-POSSUM and CR-POSSUM, as well as exponential analysis for POSSUM mortality.

## 3. Results

### 3.1. Morbidity: Real-Life Data and POSSUM Predictions

**Figure 1:**
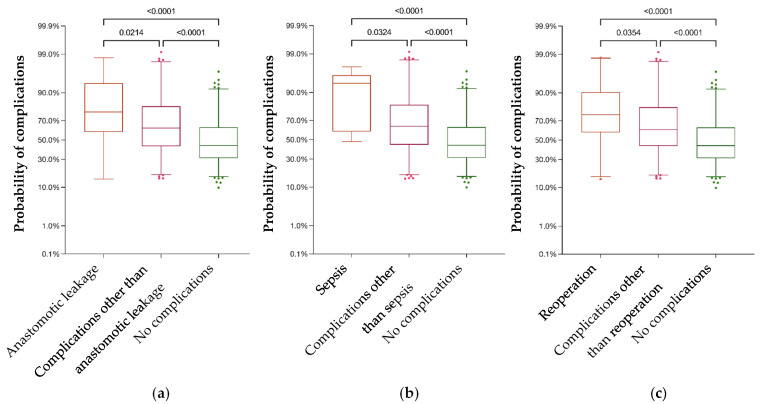
Patients suffering postoperatively from anastomotic leakage (**a**) or sepsis (**b**), as well as patients requiring reoperation (**c**), had a significantly elevated POSSUM morbidity score compared to those with other complications or without any complications, respectively.

The overall in-hospital morbidity was 47.6%, and 447 complications were registered in total, while in some cases, multiple complications per patient occurred (Table S1). The patients´ age had no significant influence on the postoperative complication rate (*p* = 0.978). Male patients and patients treated for rectal cancer had significantly more complications (*p* < 0.001; *p* = 0.016, respectively), while patients treated by laparoscopic surgery had significantly less complications (*p* = 0.017). The original POSSUM equation significantly overpredicted postoperative morbidity in our study cohorts. The average calculated morbidity was 55.33% (SD = 0.235), with a 95% confidence level reaching from 51% to 57.20%, while the actual morbidity was 47.6%. Male patients had a significantly higher predicted probability of postoperative complications with 57.33% (SD = 0.239) compared to female patients with 52% (SD = 0.225; *p* = 0.017). For each of the three age sets, significantly different complication rates were calculated. The predicted complication rates were 44.79% (SD = 0.241) for patients under 60 y, 51.57% (SD = 0.231) for patients from 61 to 70 y, and 64.14% (SD = 0.201) for patients older than 71 y. Patients with colon cancer had a significantly lower predicted complication rate with a mean value of 52.02% (SD = 0.232) compared to patients with rectal cancer with a predicted mean morbidity of 59.88% (SD = 0.232) (*p* = 0.0004). The predicted probability of 49.52% (SD = 0.219) for postoperative complications was significantly lower for patients treated by laparoscopic surgery compared to 58.05% (SD = 0.238) for patients undergoing open-approach surgery (*p* = 0.0002). Intriguingly, the predicted POSSUM morbidity for those patients suffering from anastomotic leakage (mean 73.49, SD = 0.233) was significantly higher than the scores for patients with other complications (mean 61.68, SD = 22.84), or those without complications (mean 48.05%, SD = 0.217; Figure 1a). The same observation was made for patients suffering from septicemia (mean 81.97%, SD = 0.188; Figure 1b) as well as for patients requiring reoperation (mean 71.75%, SD = 0.223; Figure 1c).

### 3.2. Prediction of Mortality by POSSUM, P-POSSUM and CR-POSSUM

**Figure 2:**
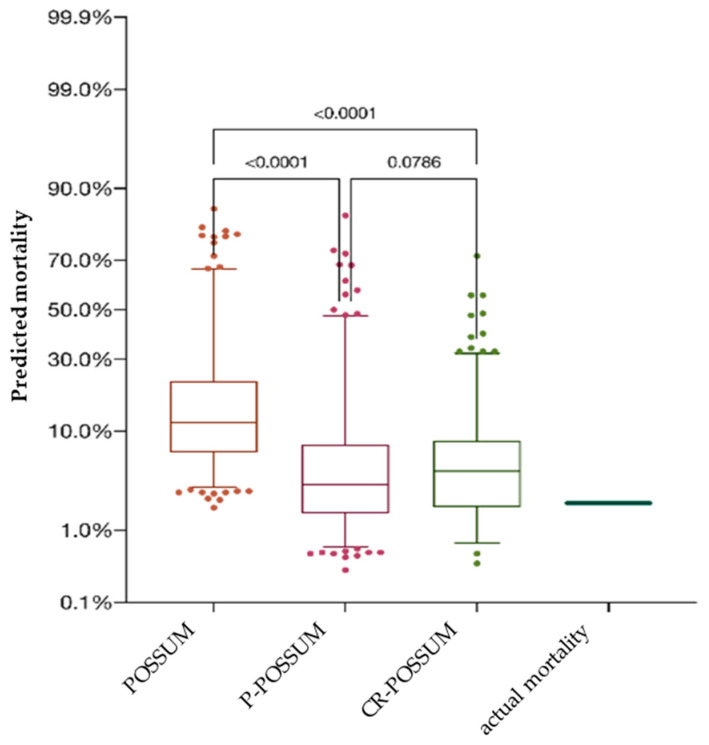
Comparison of the predicted mortality of POSSUM, P-POSSUM, and CR-POSSUM. The predicted mortality of POSSUM was significantly higher compared to the prognostic values of P-POSSUM and CR-POSSUM.

The overall in-hospital mortality of both patient-collectives combined aggregated 10 patients (2.06%), with the male-to-female ratio being 4:1. There were no significant differences regarding mortality between genders (*p* = 0.334) or age (*p* = 0.052). Moreover, mortality rates were not significantly influenced by tumor localization (colon vs. rectal cancer, *p* = 0.332) or surgery modality (open approach vs. laparoscopic, *p* = 0.78). All three prognostic scoring systems considerably overpredicted the in-hospital mortality (Figure 2).

**Figure 3:**
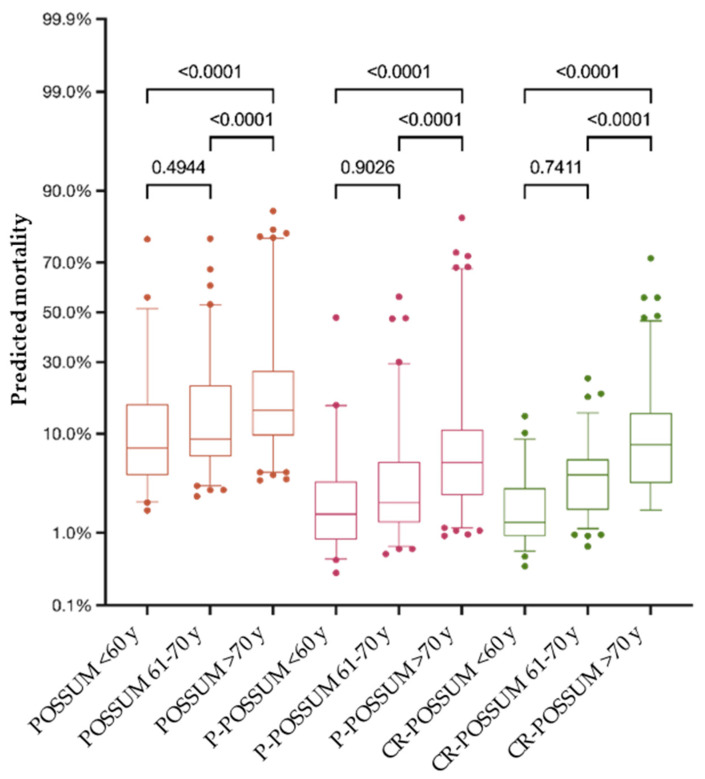
Graduating the patients by age brackets leads to a significant increase in the predicted mortality for patients older than 70 years, regardless of the scoring system applied.

The predicted mortality of POSSUM was significantly higher compared to the prognostic values of P-POSSUM and CR-POSSUM (*p* < 0.0001). The average predicted mortality by POSSUM was 17.54% (SD = 0.160), corresponding to 83.65 predicted deaths, more than nine-fold higher than the observed death rate in the study population. P-POSSUM and CR-POSSUM predicted an average mortality of 7.39% (SD = 0.116), equivalent to 35.25 deaths, and 6.98% (SD = 0.083), equivalent to 33.85 deaths, respectively. There were no significant differences between the predicted mortality rates for male and female patients calculated by POSSUM (*p* = 0.174), P-POSSUM (*p* = 0.174), and CR-POSSUM (*p* > 0.999). Remarkably, all three models prognosticated an erratic increase in mortality for patients older than 70 years (Figure 3).

**Figure 4:**
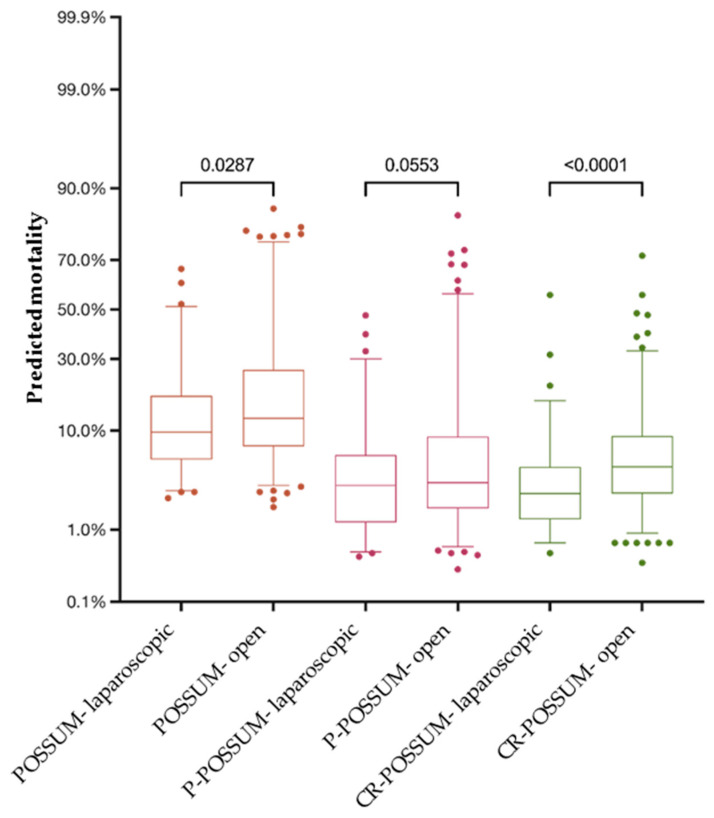
The predicted mortality for patients undergoing laparoscopic resection was significantly lower compared to patients treated by open approach in all three scoring systems.

Although the POSSUM and CR-POSSUM models predicted higher mortality for patients with rectal cancer (*p* = 0.024 and *p* = 0.001 resp.), the P-POSSUM scores for patients with colon and rectal cancer did not differ significantly (*p* = 0.445). Regarding the surgical approach, viz open vs. laparoscopic, all three scoring systems prognosticated significantly lower mortality for patients undergoing laparoscopic surgery (Figure 4).

To further specify the accuracy of the scoring systems, we calculated observed:predicted (O:E) mortality ratios after stratifying the cohort for specific risk groups and performed an exponential analysis for the POSSUM model and linear analyses for P-POSSUM and CR-POSSUM as described by Wijesinghe et al. [9] (Table 4).

Although O:E values below 1 indicate an overprediction of mortality, indicating that the quality of surgical outcome is better than expected, O:E values above 1 suggest an underprediction of mortality. The POSSUM model showed a thorough overprediction of mortality for all risk groups. The P-POSSUM model performed relatively accurately for patients with predicted mortality between 60 and 70% (O:E 1.01) and 80–90% (O:E 1.19) but vastly overpredicted mortality for all other risk groups. Likewise, the CR-POSSUM model overpredicts mortality in all risk brackets and reaches a maximal accuracy in the risk decile of 50–60% with an O:E ratio of 0.89.

### 3.3. Impact of Subscores on Observed Morbidity and Mortality

**Figure 5:**
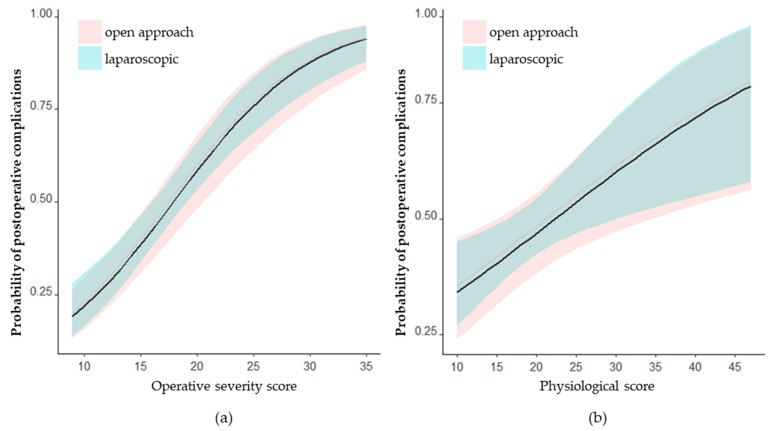
The multivariate logistic regression, in which one subscore is kept constant in each case, shows the probability of complications as a function of OS (**a**) or PS (**b**), with the standard deviation being represented by colored shading, while the median is represented by a continuous curve. The almost complete congruence of the curves indicates that the likelihood of complications depends not on the surgical approach but on the level of the individual subscores.

By an analysis of the relation of the POSSUM and P-POSSUM subscores, the PS and OS revealed a significantly higher OS for patients undergoing open-approach surgery compared to the laparoscopic subgroup (*p* < 0.001). In contrast, there were no significant differences between open approach vs. laparoscopic surgery regarding PS (*p* = 0.11). Logistic regression demonstrated a significant impact of the two subscores on the complication probability. A multivariate analysis, adjusted for the OS, demonstrated the elimination of the difference between laparoscopic and open surgery regarding the probability of postoperative complications (Figure 5a). This observation was likewise for multivariate analysis adjusted for the PS, while the scattering of the morbidity probability increased with growing PS (Figure 5b).

The logistic regression analysis demonstrated a strong correlation between the mortality probability and the PS and OS of POSSUM and P-POSSUM. Although the CR-POSSUM PS was linked to mortality, this relationship was not observed for the CR-POSSUM OS. Regarding the surgical modality (open vs. laparoscopic resection), neither the PS and OS calculated with POSSUM and P-POSSUM nor PS and OS calculated with CR-POSSUM had a significant impact on postoperative mortality (Table 5).

## 4. Discussion

The in-hospital mortality for all patients of our study combined was 2.06%, which is in accord with the 30-day mortality of 2.6% reported from a comparable colorectal cancer population of the American National Cancer Database [10]. Compared to large prospective studies, like the COLOR and COLOR II trials, which reported complication rates of 20–21% for patients with colon cancer and 37–40% for patients with rectal cancer, the observed morbidity in our population was reasonably higher. This might be attributed to the strict exclusion criteria of the aforementioned works [11,12]. Clauer et al. reported complication rates of 39.4% for colon cancer patients and 48.6% for patients with rectal cancer for a large observed German colorectal cancer population [13].

The POSSUM equation significantly overpredicted the postoperative morbidity of our study population and predicted a considerable increase in complications with increasing age. Moreover, all three prognostic models severely overpredicted mortality, especially for patients with low perioperative risk. Predominantly, these observations reflect the improvement of surgical methods on the one hand, as well as the increasing general health status of the overall population, with a forward shift in life expectancy in the recent decades on the other hand. The improvements in cancer treatments over the decades are obviously reflected by the continuously increasing 5-year survival rates for colorectal cancer [14]. One reason for this inaccuracy might be the overvaluation of the age factor in all three prediction models. A recent study from Japan showed no significant differences in the 30-day mortality after colorectal cancer surgery between patients younger than 60 years, septuagenarians, and patients above 80 years [15]. Also, Lim et al. demonstrated the safety of laparoscopic surgery for CRC in patients older than 80 y with no increase in mortality compared to patients below the age of 60 [16]. Other authors reported a decline in the postoperative mortality of colorectal cancer surgery in older patients over the recent decades, too [17,18]. Even though age is associated with malnutrition, frailty, and multimorbidity, chronological age alone is not adequate to evaluate individual fitness and health [19]. Moreover, none of the scoring systems incorporates other risk factors known to be associated with morbidity and/or mortality, such as diabetes, smoking status, alcohol abuse, and obesity [20,21,22,23]. The POSSUM and P-POSSUM physiological scores neglect a differentiation between distinct cardiovascular medications, potentially contributing to (cardiovascular) risk overestimation. Although angina pectoris, myocardial infarction, and congestive heart failure are associated with increased mortality after colorectal surgery, the most common cardiovascular disease, hypertension, is neither associated with mortality nor with postoperative complications [24,25]. Since 31.6% of the adult German population suffers from hypertension, and more than half of these patients are treated with antihypertensive medication, an “overscoring” of the study population seems likely [26]. On the contrary, COPD and atrial fibrillation have been identified as risk factors for mortality and postoperative complications in colorectal surgery in several studies [24,25,27]. The category “blood loss” diminishes the objectivity of the OS since even experienced surgeons tend to overestimate small and underestimate large blood losses [28]. Moreover, blood loss estimation formulae, based on different hematological indices, are also incapable of correctly determining the actual blood loss [29]. Not least of all, neither of the scoring systems can measure and regard the aspect of surgical skill and experience of the individual surgeon.

Ultimately, our study design has several limitations. Due to the relatively long observation period, the significance and utility of our dataset may have been influenced by the increasing adoption of laparoscopic techniques, improvements in stapling devices, and the implementation of enhanced recovery after surgery. The average hospital length of stay in Germany at the end of the observation period was 16 days [30]. Therefore, it cannot be completely ruled out that post-hospital events are not recorded, and thus, hospital mortality and 30-day mortality may not fully align. Since our dataset is limited to two tertiary centers in a defined geographical location (Northern Germany), the applicability of the results to a broader population remains questionable. Indeed, findings on the accuracy of the different scoring systems differ between populations. Although all three scoring systems were able to reliably predict mortality or morbidity in an Australasian population of colorectal cancer patients, data from Brazil showed a precise prediction of mortality by P-POSSUM but not by POSSUM [31,32]. In a large Chinese cohort of 903 patients undergoing surgery for colon and rectal cancers from 1992 to 2005, all three scoring systems significantly overestimated mortality [33]. A more recent evaluation of the reliability of POSSUM, P-POSSUM, and CR-POSSUM for the prediction of mortality in patients undergoing colorectal surgery in a single UK hospital found an overestimation of mortality by all three scoring systems [34]. In recent decades, multiple alternative approaches to the estimation of perioperative morbidity and mortality have been developed and validated. The Association of Coloproctology of Great Britain and Ireland (ACPGBI)-score was introduced in 2003 under the premise of a simpler applicability by assessing only five operative variables: age, cancer resection, American Society of Anesthesiology grade, Dukes’ stage, and operative urgency [35]. Ferjani et al. tested the ACPGBI model alongside the three POSSUM scoring systems in a large population undergoing colorectal resection. Despite the ACPGBI score being a significantly better predictor of overall mortality compared to POSSUM and P-POSSUM, it had no advantage over the predictive accuracy of CR-POSSUM [36]. The Japanese Estimation of Physiologic Ability and Surgical Stress (E-PASS) and its modified version, mE-PASS for colorectal surgery, consider the heart and pulmonary disease, diabetes mellitus, performance status, and American Society of Anesthesiologists physiological status on the one hand, as well as blood loss to body weight ratio, operation time and extend of skin incision on the other hand [37]. Although the discriminatory power of mE-PASS regarding postoperative mortality rates was superior to POSSUM, P-POSSUM, and CR-POSSUM in a Japanese patient population, mE-PASS performed “equally imprecise” when applied to a British cohort [34,38]. The regression model implemented by the Association Francaise de Chirurgie (AFC) to predict in-hospital mortality in colorectal surgery is based on the multivariate analysis of the four variables (urgent surgery, loss of body weight greater than 10%, neurologic comorbidity, and age older than 70 years) without the application of weighting or an additional equation [39]. The Identification of Risk in Colorectal Surgery (IRCS) score was developed in the Netherlands and validated for a Spanish colorectal cancer population. Five variables—age, disease stage, urgency, and cardiac and pulmonary function—are weighted and put into an equation [40]. A direct comparison of CR-POSSUM, IRCS, and AFC scores for the analysis of a large Spanish colorectal cancer cohort revealed similar accuracy of the first two models, while the AFC performed less precisely [41]. Although previous models aimed for a reduction of variables and weighting, the ACS-NSQIP calculator takes a new approach by assessing 21 preoperative risk factors as well as the subjective surgeon adjustment score, allowing a precise and easy calculation of perioperative complications via a web-based form [42]. With the advent of artificial intelligence, the processing of large datasets with numerous variables is becoming increasingly easier. Van den Bosch et al. recently demonstrated that machine learning applied to a surgical audit of colorectal cancer patients outperformed conventional scores in predicting 30-day postoperative mortality [24]

## 5. Conclusions

Despite the relative inaccuracy, the POSSUM score was able to identify patients at risk of severe complications, like anastomotic leakage, sepsis, and the need for reoperation. Since the PS of POSSUM, P-POSSUM, and CR-POSSUM, and the OS of the POSSUM and P-POSSUM, are significantly associated with mortality, they might be nevertheless useful to identify patients at risk of dying in the field of colorectal cancer surgery. However, one major flaw of POSSUM and its variations is the lack of variables not until after the intervention has effectively been performed (OS). Therefore, none of the scoring systems should be taken into account when counseling patients regarding perioperative within the context of informed consent. Thirty years after the introduction of the POSSUM system, due to the increasing digitization of health data and advancements in machine learning, we can expect a more reliable and precise tool for the prediction of perioperative complications and mortality in the near future.

## Figures and Tables

**Table 1:** POSSUM and P-POSSUM scoring system.

Scoring	1	2	4	8
Physiological Score				
Age (years)	≤60	61–70	≥71	
Cardiac	No failure	Drug treatment	Edema/Warfarin	Raised jugular vein pressure
Chest radiograph	Normal		Slight cardiomegaly	Cardiomegaly
Respiratory	No dyspnea	Dyspnea on exertion	Limiting dyspnea (one flight)	Dyspnea at rest (rate > 30/min)
Chest radiograph		Mild COPD	Moderate COPD	Fibrosis/consolidation
Syst. blood pressure (mmHg)	110–130	131–170/100–109	≥171/90–99	≤89
Pulse rate (/min)	50–80	81–100/40–49	101–120	≥121/≤39
Glasgow Coma Scale	15	12–14	9–11	≤8
Urea (mmol/L)	≤7.5	7.6–10	10.1–15	≥15.1
Hemoglobin (g/L)	13–16	11.5–12.9/16.1–17.0	10.0–11.4/17.1–18.0	≤9.9/≥18.1
White cell count (×10^12^/L)	4–10	10.1–20.0/3.1–3.9	≥21.1/≤3	
Sodium (mmol/L)	≥136	131–135	126–130	≤125
Potassium (mmol/L)	3.4–5	3.2–3.4/5.1–5.3	2.9–3.1/4.4–5.9	≤2.8/≥6
Electrocardiogram	normal		Atrial fibrillation (60–90/min)	Any other abnormality
Operative Severity Score				
Operative Severity *	minor	moderate	major	Major+
Multiple procedures	1		2	>2
Total blood loss (mL)	≤100	101–500	501–999	≥1000
Peritoneal soiling	none	Minor (serous fluid)	Local pus	Free bowel content /pus/blood
Presence of Malignancy	none	Primary only	Nodal metastases	Distant metastases
Mode of surgery	elective		Emergency surgery < 24 after admission	Emergency surgery < 2 h after admission

* Major: any laparotomy, any bowel resection; major+: abdominoperineal rectum resection. None of the possible procedures for colorectal cancer could be classified as minor or moderate.

**Table 2:** CR-POSSUM scoring system.

Scoring	1	2	3	4	8
Physiological Score					
Age group	≤60		61–70	71–80	≥81
Cardiac failure	None or mild	moderate	severe		
Syst. RR (mmHg)	100–170	>170/90–99	<90		
Pulse	40–100	101–120	>120/<40		
Urea (mmol/L)	≤10	10.1–15	>15		
Hemoglobin (g/dL)	13–16	10–12.9/16.1–18	<10/>18		
Operative Severity Score					
Operative Severity *	Minor		Intermediate	Major	Complex major
Peritoneal soiling	None/serous fluid	Local pus	Free Pus or feces		
Operative Urgency	Elective		Urgent		Emergency
Cancer staging	No cancer or Dukes’ A–B	Dukes’ C	Dukes’ D		

* Major: right/left/extended right/extended left hemicolectomy, sigmoid resection, transverse colectomy; complex major: (lower) anterior resection, subtotal or total colectomy, abdominoperineal rectum resection. Minor or intermediate procedures, like small proctologic procedures and transanal resection of tumors, were excluded from our study.

**Table 3:** POSSUM, P-POSSUM, and CR-POSSUM equations.

	POSSUM	P-POSSUM	CR-POSSUM
Mortality	$\ln\left[\frac{R}{\left(1-R\right)}\right]=$ −7.04 + (0.13 × PS) + (0.16 × OSS)	$\ln\left[\frac{R}{\left(1-R\right)}\right]=-$9.37 + (0.19 × PS) + (0.15 × OSS)	$\ln\left[\frac{R}{\left(1-R\right)}\right]=-$9.167 + (0.338 × PS) + (0.308 × OSS)
Morbidity	$\ln\left[\frac{R}{\left(1-R\right)}\right]=-$5.91 + (0.16 × PS) + (0.19 × OSS)		

**Table 4:** O:E ratios of POSSUM, P-POSSUM and CR-POSSUM.

	Exponential Analysis				Linear Analysis							
	POSSUM				P-POSSUM				CR-POSSUM			
risk [%]	n	O	E	O:E	n	O	E	O:E	n	O	E	O:E
0–100	477	9	83.66	0.11	386	3	12.56	0.24	381	4	14.17	0.28
10–100	266	8	70.4	0.11	52	2	7	0.29	77	3	10.68	0.28
20–100	142	6	52.8	0.11	15	0	3.72	0.00	12	2	2.797	0.72
30–100	77	4	37	0.11	8	0	2.85	0.00	9	0	3	0.00
40–100	48	4	27	0.15	7	0	3.18	0.00	3	0	1.36	0.00
50–100	29	4	18.57	0.22	3	1	1.65	0.61	2	1	1.12	0.89
60–100	16	4	11.63	0.34	3	2	1.98	1.01	0	0		
70–100	9	4	7.1	0.56	2	0	1.45	0.00	1	0	0.715	0.00
80–100	2		1.65		1	1	0.84	1.19	0	0		
90–100	0				0				0	0		

**Table 5:** Impact of PS and OS on postoperative morbidity and mortality.

	Odds Ratio	2.5% CI	97.5% CI
Morbidity			
PS	1.0542	1.017	1.094
OS	1.176	1.125	1.232
Open vs. laparoscopic	1.063	0.691	1.637
Mortality			
PS	1.197	1.092	1.322
OS	1.148	1.014	1.303
Open vs. laparoscopic	0.524	0.105	2.909
CR-PS	1.412	1.144	1.755
CP-OS	1.105	0.824	1.472
Open vs. laparoscopic	0.649	0.159	2.839

## Data Availability

The data presented in this study are available on request from the corresponding author. The data are not publicly available due to privacy and confidentiality considerations.

## Supplementary Materials

The following supporting information can be downloaded at: https://www.mdpi.com/article/10.3390/jcm13010173/s1, Figure S1: Patient selection and study design; Table S1: Mortality, morbidity and registered complications.
