# Supplementary material for: More than 30 Years of POSSUM: Are Scoring Systems Still Relevant Today for Colorectal Surgery?
Source: J Clin Med. 2023 Dec 28;13(1):173. doi: 10.3390/jcm13010173 (PMC10779462; doi:10.3390/jcm13010173)
Supplement: Supplementary file 1 [file jcm-13-00173-s001.zip › jcm-2742312-supplementary.pdf]

Figure S1: Patient selection and study design

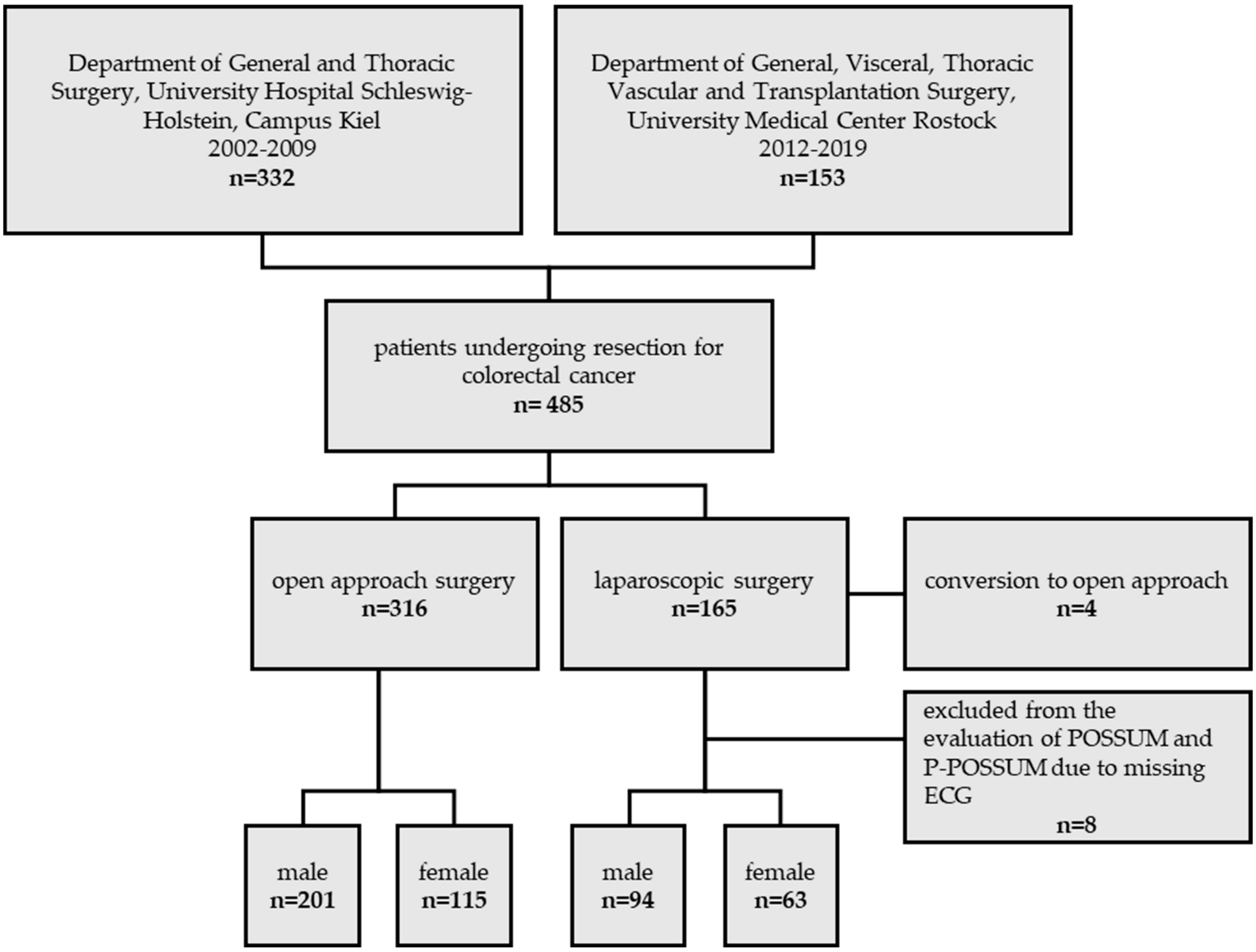

Supplementary Figure S1. The retrospective analysis of two independent German patient populations undergoing surgery for colorectal cancer included 332 cases of the Department of General and Thoracic Surgery, University Hospital Schleswig-Holstein, Campus Kiel, Kiel in a period from 2002 to 2009 and 153 laparoscopic colorectal resections of the Department of General, Visceral, Vascular and Transplantation Surgery, University Medical Center Rostock in a period from 2012 to 2019. Conversion from laparoscopy to laparotomy was necessary in four cases, which were excluded from comparison of laparoscopic vs. open approach. Eight patients from the laparoscopic cohort were excluded from the analysis by POSSUM and P-POSSUM due to a missing electrocardiogram.

Supplementary Table S1: Mortality, morbidity and registered complications

**Mortality**	**10 (2.06 %)**
Colon cancer	4 (1.41 %)
Rectal cancer	6 (2.97 %)
**Complications in total (more than one per patient possible)**	**447**
Complication rate	47.6 %
→Colon cancer	41.17
→Rectal cancer	53,46 %
**Enumeration of complication subtypes and their percentage portion of total complications**	
Reduced gastrointestinal motility	91 (18.76 %)
Secondary wound healing	61 (12,58 %)
Reoperation for any cause	51 (10.52 %)
Anastomotic leakage	39 (8.04 %)
Urinary tract infection requiring antibiotic treatment	34 (7.01 %)
Hospital acquired pneumonia	30 (6.19 %)
Hemorrhage of any kind	15 (3.09 %)
Abdominal wound dehiscence	14 (2.89 %)
Renal dysfunction	14 (2.89 %)
Splenic injury	13 (2.68 %)
Peritonitis requiring reoperation	13 (2.68 %)
Sepsis	13 (2.68 %)
Lesions of the small intestines	8 (1.65 %)
Intestinal ischemia	7 (1.44 %)
Intraabdominal abscess	6 (1.24 %)
Deep vein thrombosis	6 (1.24 %)
Postoperative hemorrhage requiring reoperation	6 (1.24 %)
Pulmonary embolism	4 (0.82 %)
Urinary bladder injury	4 (0.82 %)
Cardiopulmonary resuscitation	3 (0.62 %)
Hypertensive crisis	3 (0.62 %)
Anastomotic stenosis	3 (0.62 %)
Myocardial infarction	2 (0.41%)
Liver injury	2 (0.41%)
Hollow organ perforation	2 (0.41%)
Recurrent laryngeal nerve injury	1 (0.21 %)
Pancreatic injury	1 (0.21 %)
Ureteral injury	1 (0.21 %)
